# Incidence of Postoperative Infection Following Simultaneous Bilateral Knee Arthroplasty: A Systematic Review and Meta-Analysis

**DOI:** 10.7759/cureus.54117

**Published:** 2024-02-13

**Authors:** Bashar Reda, Raed Sharaf

**Affiliations:** 1 Orthopedic Surgery, College of Medicine, King Abdulaziz University, Jeddah, SAU; 2 College of Medicine, King Abdulaziz University, Jeddah, SAU

**Keywords:** surgical site infection, infection, knee replacement, total knee arthroplasty, bilateral, simultaneous

## Abstract

Total knee arthroplasty is one of the most common orthopedic procedures. Simultaneous bilateral knee arthroplasty involves performing total knee arthroplasty on both knees in a single anesthetic session.

This systematic review and meta-analysis followed the guidelines of the Preferred Reporting Items for Systematic Reviews and Meta-Analyses 2020. A primary search was performed using PubMed, EBSCO, Scopus, Web of Science, Clarivate, and Google Scholar databases. Quantitative data synthesis was performed using MedCalc® Statistical Software version 20.115 to determine the pooled prevalence of the infection among patients who underwent simultaneous bilateral knee arthroplasty. The Newcastle-Ottawa Scale was used to assess study quality.

We included 30 studies in our quantitative data synthesis, with a total population of 118,502 patients (237,004 knees). The pooled prevalence of superficial infection, deep infection, and unspecified surgical site infection was estimated to be 0.86% (95% confidence interval: 0.62-1.13%), 0.84% (95% confidence interval: 0.64-1.05%), and 1.18% (95% confidence interval: 0.45-2.27%), respectively. There was significant heterogeneity (I2 >50%) in all analyses, and inspection of funnel plots revealed a symmetrical distribution of plotted data.

We found that the infection rates following simultaneous bilateral knee arthroplasty were relatively low but heterogeneous, as the data showed marked variability. Superficial infections were more common than deep infections; however, there was a small difference in their prevalence. Furthermore, the reliability of our findings was limited owing to significant heterogeneity.

## Introduction and background

The frequency of performing total knee arthroplasty (TKA) surgeries is constantly increasing in developed nations with an aging population and increasing obesity. The National Joint Registry (NJR) documented 102,177 primary knee replacement procedures in 2017, an increase of 3.7% from 2015. Additionally, 96.2% of these procedures were performed on patients with osteoarthritis. According to a data analysis conducted by the NJR and the Office of National Statistics, primary TKAs in England and Wales will increase by 117% between 2012 and 2030. Typically, 75-85% of patients report being satisfied (or "very satisfied") with the results of their surgeries, whereas the remaining 15.25% were dissatisfied (or "very dissatisfied) [1].

Interestingly, the satisfaction level after TKA is much lower than that after total hip arthroplasty, which emphasizes the necessity for additional investigation into the causes behind this [2,3]. Patient satisfaction might vary for various reasons and depends on the techniques used to evaluate the results, the preoperative expectations of the patients, or the general preoperative state. As it is convenient for surgeons, the National Health Service progression reports on TKA are now based on the results reported by the patients using the Oxford Knee Score. The approaches utilized after TKA for the same are outlined in the following section, along with their benefits and drawbacks.

Bilateral knee joint symptoms are common in individuals with severe end-stage degenerative joint disease and require joint replacement in both knees [4,5]. These patients have three surgical options: bilateral arthroplasty utilizing one surgical team while the patient is under anesthesia, simultaneous arthroplasty of both knees using two surgical teams, and staged surgery with a set duration between the two surgeries. However, there is disagreement about whether bilateral knee arthroplasty should be conducted simultaneously or in stages [6].

TKA is a safe and successful procedure, although periprosthetic joint infection (PJI), deep infections, and other postoperative sequelae remain major issues. PJI plays a significant role in implant failure and revision arthroplasty [7]. Compared to unilateral operations, simultaneous bilateral knee arthroplasty (simBTKA) is associated with a longer duration of hospital stay. The development of deep infections has been reported to predispose patients to longer operating times [8]. However, as of now, the results of previous studies examining the impact of simBTKA on PJIs have been inconsistent [9-11]. Therefore, it is still crucial to determine if these longer operations have a higher incidence of infection problems than staged and unilateral arthroplasties. Therefore, with this meta-analysis, we aimed to estimate the burden of postoperative infections following simBTKA.

This systematic review and meta-analysis were conducted in accordance with the 2020 PRISMA (Preferred Reporting Items for Systematic Reviews and Meta-Analysis) guidelines [12].

The search strategy for these studies was compiled using searches of PubMed, EBSCO, Scopus, and Web of Science through Clarivate and Google Scholar databases. We searched databases using keywords, MeSH terms, and the Boolean operators AND and OR. The search keywords included bilateral total knee arthroplasty, bilateral TKA, knee replacement, knee arthroplasty, infect, sepsis, SSI, surgical site infection, deep infection, superficial infection, simultaneous, simultaneous BTKA, single anesthetic, and simBTKA. In addition to database searches, we manually searched for articles via Google. Since TKA was first identified in the 1970s and 1980s, no language constraints were used, and publications from January 1970 to the present were searched.

Three writers assessed the titles, abstracts, and full texts of the findings to assess whether the search results fulfilled the criteria for inclusion in this systematic review. Conflicts between the two authors were resolved by discussion with the third author or by reaching an agreement. An email was used to contact the authors of the publications if further information on potential studies was required. All information relevant to the research subject was obtained from the included articles and entered into Microsoft Excel (Microsoft Corp., Redwood City, Calif., USA).

The Rayyan Intelligent Systematic Reviews website was used to manage the primary search results and eliminate duplicates. After performing title, abstract, and full-text screening, Microsoft Excel was used to extract data from the included studies.

The quality of the studies was assessed by two authors using the Newcastle-Ottawa Scale (NOS). The values for the quality evaluation items ranged from 0 to 9. A study was considered high quality if it earned seven or more.

For quantitative data synthesis, MedCalc® Statistical Software version 20.115 (MedCalc Software Ltd., 2022) was used. Data on superficial, deep, and unspecified infections were included in a proportional random effects meta-analysis, on which forest and funnel plots were constructed. The proportion of inter-study heterogeneity was measured using Higgin's I2 test, with a cut-off threshold for considerable heterogeneity set at I2>50%. Funnel plots were used to visually analyze publication bias with a cut-off point of a p-value of 0.1 or less.

## Review

Results

**Figure 1:**
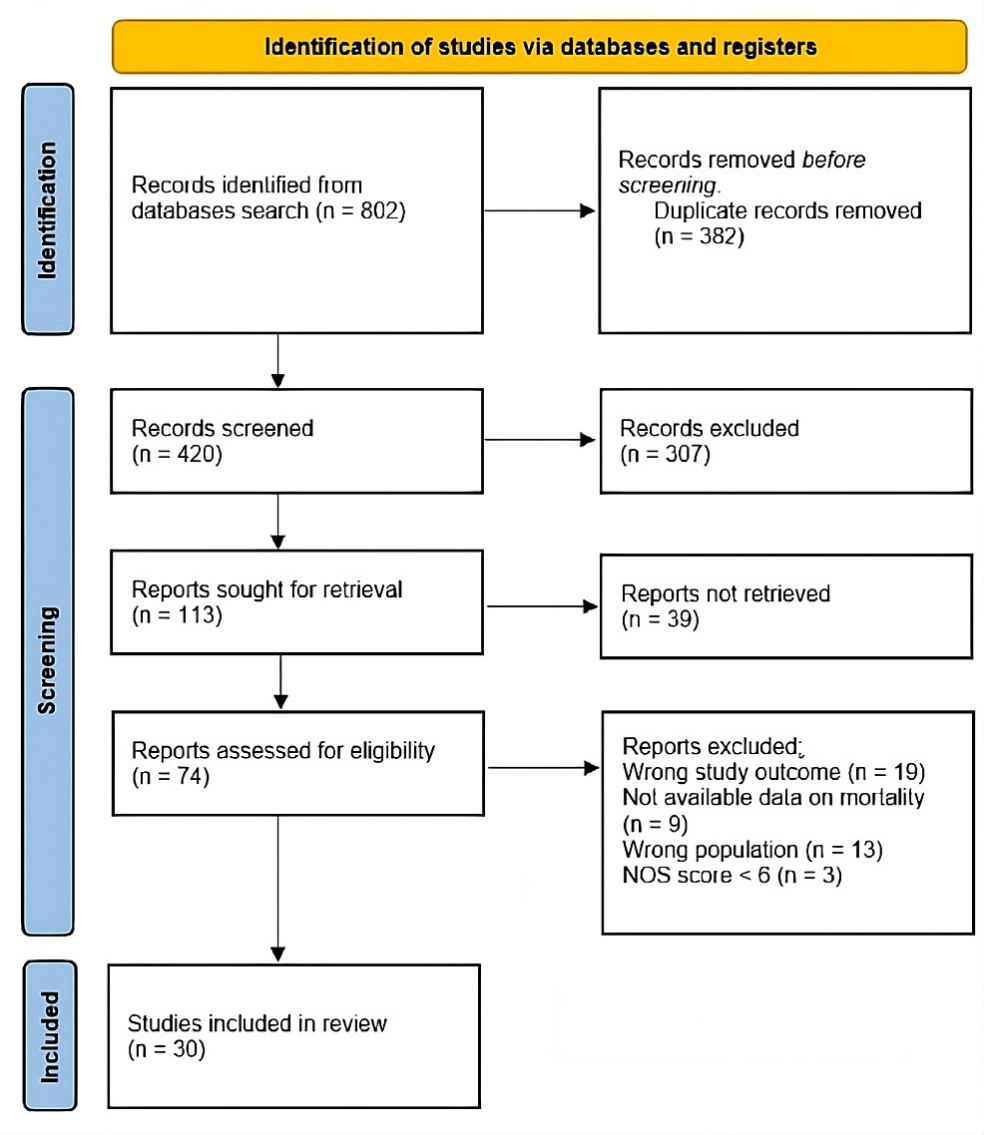
PRISMA flow chart summarizing the search and screening processes

In total, 802 studies were identified by using the aforementioned electronic databases. The exclusion of 382 studies due to duplicate detection and removal left 420 studies available for enrollment during the title and abstract screening. After screening titles and abstracts, 307 studies were eliminated. Thirty studies were included in the analysis after the full texts of the remaining 74 studies were evaluated. The search and study selection procedures are summarized in Figure 1.

Characters of the included studies

After completing the search and screening of the primarily extracted studies, 30 studies were finally included for quantitative data synthesis: 8, 10, 13, 14, 15, 16, 24, 25, 26, 27, 28, 29, 30, 31, 32, 33, 34, 35, 36, 37, 38, 39, 40, 42, 43, 44, 45, 46, 47, 48. Twelve of the included studies were conducted in the USA: 8, 10, 14, 24, 26, 29, 32, 34, 38, 39, 40, 43, 44, 46, and 47. As shown in Table 1, the total population of each study ranged from 36 patients [13] to 27,301 patients [14], whereas the overall total population was 118,502 patients (237,004 knees). The proportion of males ranged from 5% to 57.9% [15-17] . The safety of simBTKA has been thoroughly investigated [18-20]. Additionally, complications such as infection, in addition to costs compared to anesthesia once or twice [21-23].

**Table 1:** Characters of the included studies

Study	Design	Country	Total population	Males (%)	Mean age, y	NOS
Bini et al., 2014 [24]	Retrospective	USA	1230	41.60%	66	6
Bohm et al., 2016 [25]	Retrospective	Canada	6349	41%	64	7
Bolognesi et al., 2013 [26]	Retrospective	USA	8307	22%	73.3	6
Chan et al., 2009 [16]	Retrospective	UK	159	57.90%	66	7
Chen et al., 2013 [27]	Prospective	Singapore	124	26.60%	62.9	6
Chua et al., 2018 [28]	Retrospective	Australia	23136	53.80%		7
Courtney et al., 2014 [29]	Retrospective	USA	103	33%	59.4	6
Feng et al., 2019 [30]	Prospective	China	39	15.40%	64.9	7
Gill et al., 2020 [31]	Retrospective	Australia	122	37.70%	70.6	7
Hadley et al., 2017 [32]	Retrospective	USA	371	30%	63.9	7
Hooper et al., 2009 [33]	Retrospective	New Zealand	1012		65	8
Houdek et al., 2017 [34]	Retrospective	USA	94	57%	52.2	8
Hutchinson et al., 2006 [35]	Prospective	Australia	438	56%	67	7
Lindberg-Larsen et al., 2015 [36]	Retrospective	Denmark	157	47.10%	64	7
Lindberg-Larsen et al., 2019 [37]	Prospective	Germany	232	53.40%	64.6	8
Liu & Chen, 1998 [15]	Retrospective	China	64	5%	66.7	6
Ma et al., 2015 [13]	Retrospective	China	36	41.70%	65.6	7
Meehan et al., 2011 [10]	Retrospective	USA	11445	46.10%	67.2	8
Memtsoudis et al., 2009 [38]	Retrospective	USA	25179		66	7
Namba et al., 2012 [39]	Retrospective	USA	324			7
Poultsides et al., 2013 [8]	Retrospective	USA	2825	37.60%	65.2	8
Ritter et al., 2003 [40]	Retrospective	USA	2050	44.20%	69.9	6
Seo JG et al., 2014 [41]	Retrospective	South Korea	420			
Seol et al., 2016 [42]	Retrospective	South Korea	759	5.70%	68.3	7
Sheth et al., 2016 [43]	Retrospective	USA	3933	42.70%	64.9	8
Sobh et al., 2018 [44]	Retrospective	USA	225	48%	61	6
Spicer et al., 2013 [45]	Retrospective	Canada	373	29%	69.1	7
Triantafyllopoulos et al., 2016 [46]	Retrospective	USA	1808	51%	56.3	8
Tsay et al., 2019 [14]	Retrospective	USA	27301	43.20%	65.8	8
Wyles et al., 2019 [47]	Retrospective	USA	188	42%	61	8
Yoon et al., 2010 [48]	Retrospective	South Korea	119	5.90%	70	7

Superficial infection following simBTKA

**Figure 2:**
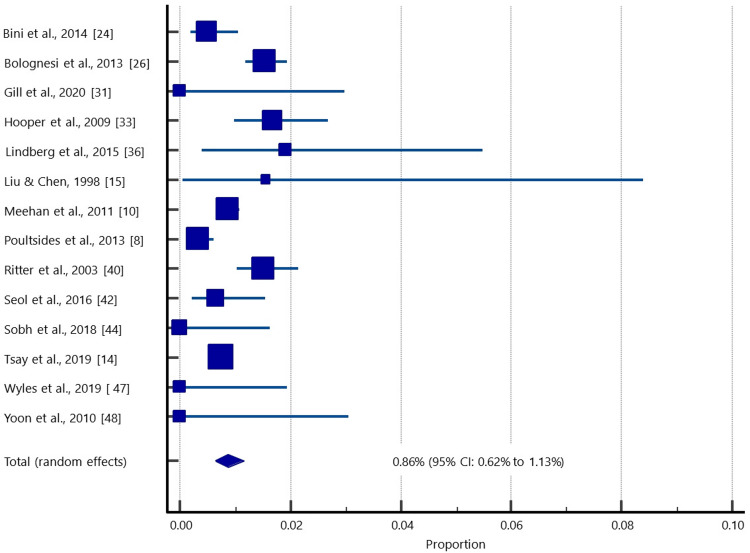
A forest plot showing the pooled superficial infection rate in patients who underwent simBTKA

Data from 52,016 patients (104,032 knees) were enrolled for quantitative data synthesis to estimate the pooled prevalence of superficial infection following simBTKA (Table 2; Figure 2). The pooled prevalence of superficial infection was estimated to be 0.86% (95% confidence interval [CI]: 0.62%-1.13%).

**Table 2:** Quantitative superficial infection data from the included studies

Study	Sample size	Proportion (%)	95% CI	Weight (%)
Bini et al., 2014 [24]	1230	0.488	0.179 to 1.059	8.98
Bolognesi et al., 2013 [26]	4519	1.527	1.190 to 1.928	12.2
Gill et al., 2020 [31]	122	0	0.000 to 2.978	2.1
Hooper et al., 2009 [33]	1012	1.68	0.982 to 2.676	8.33
Lindberg-Larsen et al 2015 [36]	157	1.911	0.396 to 5.483	2.59
Liu & Chen, 1998 [15]	64	1.562	0.0396 to 8.401	1.2
Meehan et al., 2011 [10]	11445	0.874	0.711 to 1.062	13.28
Poultsides et al., 2013 [8]	2825	0.319	0.146 to 0.604	11.29
Ritter et al., 2003 [40]	2050	1.512	1.030 to 2.140	10.5
Seol et al., 2016 [42]	759	0.659	0.214 to 1.531	7.33
Sobh et al., 2018 [44]	225	0	0.000 to 1.626	3.43
Tsay et al., 2019 [14]	27301	0.755	0.655 to 0.864	13.74
Wyles et al., 2019 [47]	188	0	0.000 to 1.943	2.99
Yoon et al., 2010 [48]	119	0	0.000 to 3.052	2.06
Total (random effects)	52016	0.857	0.620 to 1.132	100

Deep infection following simBTKA

**Figure 3:**
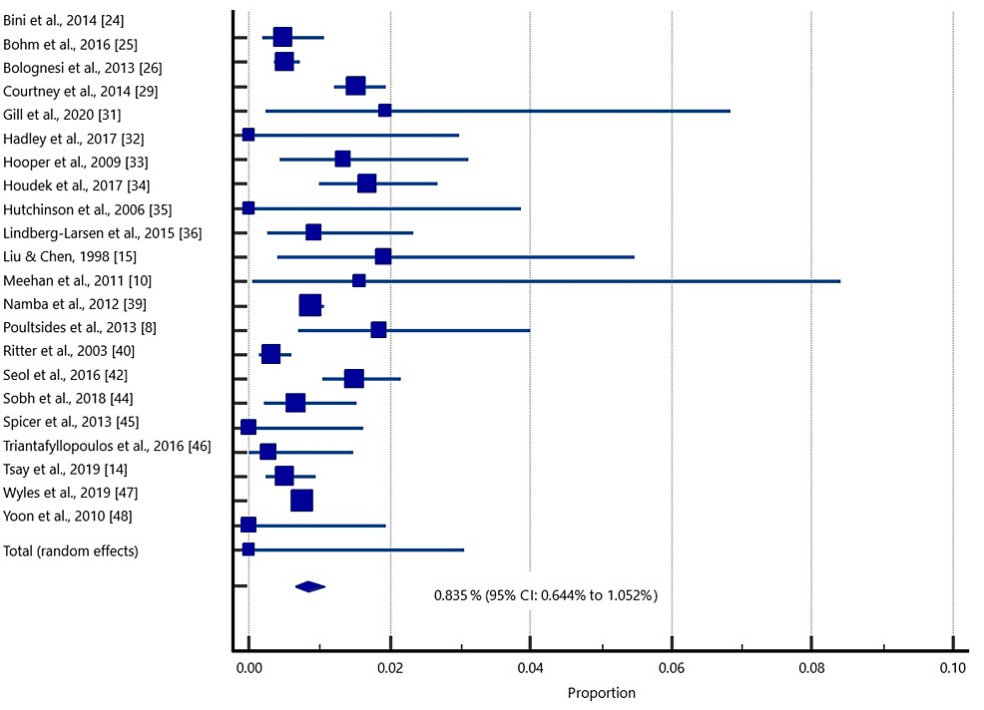
A forest plot showing the pooled deep infection rate in patients who underwent simBTKA

For quantitative data synthesis to determine the pooled prevalence of deep infection following simBTKA, data from 61,876 patients (123,752 knees) were used (Table 3; Figure 3). The estimated overall prevalence of deep infection was 0.84% (95%, CI: 0.64%-1.05%).

**Table 3:** Quantitative deep infection data from the included studies

Study	Sample size	Proportion (%)	95% CI	Weight (%)
Bini et al., 2014 [24]	1230	0.488	0.179 to 1.059	6.13
Bohm et al., 2016 [25]	6349	0.504	0.345 to 0.711	8.85
Bolognesi et al., 2013 [26]	4519	1.527	1.190 to 1.928	8.49
Courtney et al., 2014 [29]	103	1.942	0.236 to 6.839	1.2
Gill et al., 2020 [31]	122	0	0.000 to 2.978	1.38
Hadley et al., 2017 [32]	371	1.348	0.439 to 3.117	3.26
Hooper et al., 2009 [33]	1012	1.68	0.982 to 2.676	5.67
Houdek et al., 2017 [34]	94	0	0.000 to 3.848	1.1
Hutchinson et al., 2006 [35]	438	0.913	0.249 to 2.322	3.64
Lindberg-Larsen et al., 2015 [36]	157	1.911	0.396 to 5.483	1.71
Liu & Chen, 1998 [15]	64	1.562	0.0396 to 8.401	0.78
Meehan et al., 2011 [10]	11445	0.874	0.711 to 1.062	9.29
Namba et al., 2012 [39]	324	1.852	0.683 to 3.987	2.97
Poultsides et al., 2013 [8]	2825	0.319	0.146 to 0.604	7.81
Ritter et al., 2003 [40]	2050	1.512	1.030 to 2.140	7.24
Seol et al., 2016 [42]	759	0.659	0.214 to 1.531	4.96
Sobh et al., 2018 [44]	225	0	0.000 to 1.626	2.28
Spicer et al., 2013 [45]	373	0.268	0.00679 to 1.485	3.28
Triantafyllopoulos et al., 2016 [46]	1808	0.498	0.228 to 0.943	6.98
Tsay et al., 2019 [14]	27301	0.755	0.655 to 0.864	9.64
Wyles et al., 2019 [47]	188	0	0.000 to 1.943	1.98
Yoon et al., 2010 [48]	119	0	0.000 to 3.052	1.36
Total (random effects)	61876	0.835	0.644 to 1.052	100

Unspecified surgical site infection (SSI) rates

**Figure 4:**
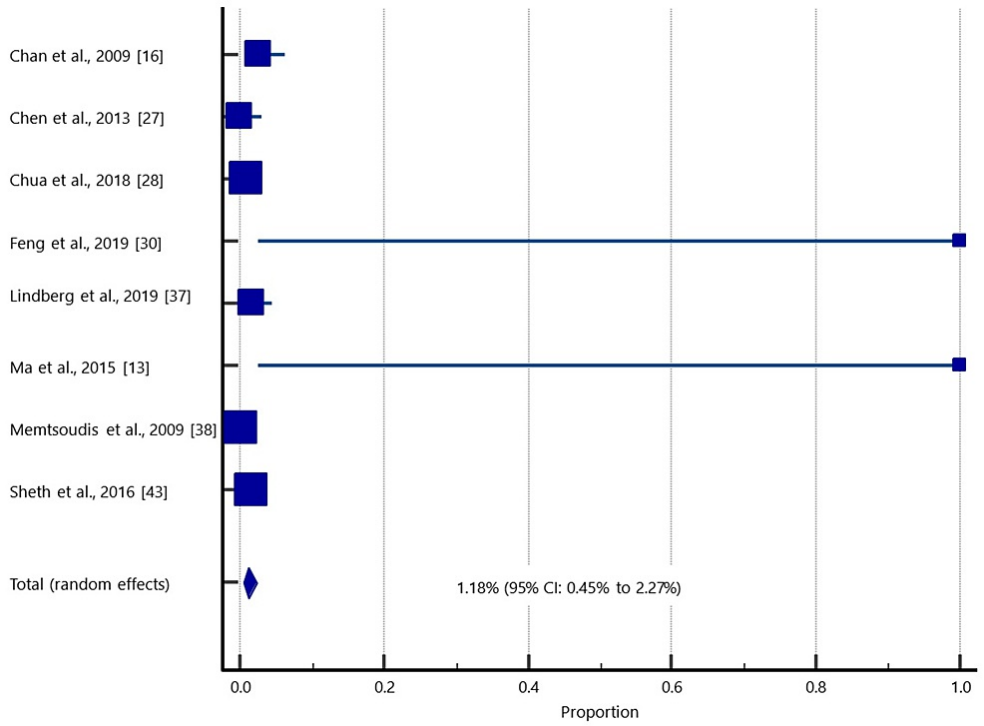
A forest plot showing the pooled unspecified SSI rate in patients who underwent simBTKA

Eight studies did not specify the type of infection. Data comprising 52,765 patients (105,530 knees) were used to estimate the pooled prevalence of infection (Table 4; Figure 4), which was found to be 1.18% (95%, CI: 0.45%-2.27%).

**Table 4:** Quantitative, unspecified SSI data from the included studies

Study	Sample size	Proportion (%)	95% CI	Weight (%)
Chan et al., 2009 [16]	159	2.516	0.690 to 6.316	12.25
Chen et al., 2013 [27]	124	0	0.000 to 2.931	10.98
Chua et al., 2018 [28]	23136	1.042	0.915 to 1.181	20.81
Feng et al., 2019 [30]	1	100	2.500 to 100.000	0.36
Lindberg-Larsen et al., 2019 [37]	232	1.724	0.472 to 4.355	14.08
Ma et al., 2015 [13]	1	100	2.500 to 100.000	0.36
Memtsoudis et al., 2009 [38]	25179	0.147	0.103 to 0.202	20.82
Sheth et al., 2016 [43]	3933	1.602	1.233 to 2.045	20.33
Total (random effects)	52765	1.182	0.445 to 2.265	100

Test for heterogeneity and publication bias

**Figure 5:**
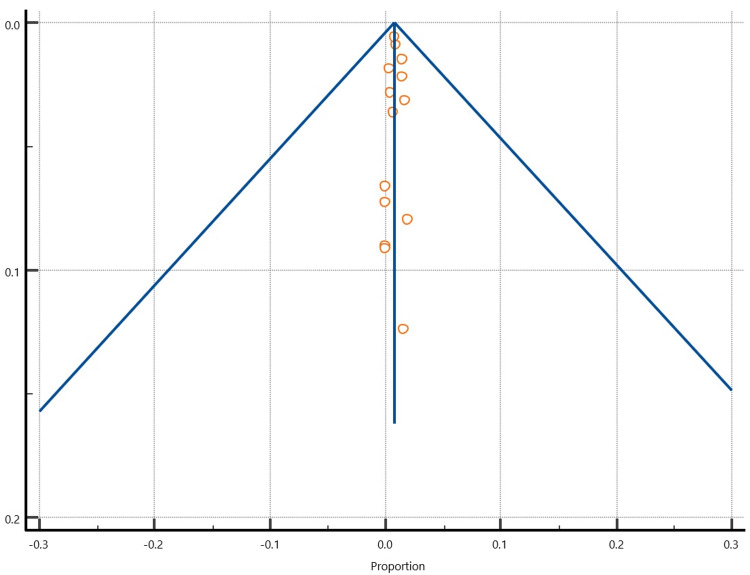
A funnel plot showing the symmetrical distribution of the plotted superficial infection data

**Figure 6:**
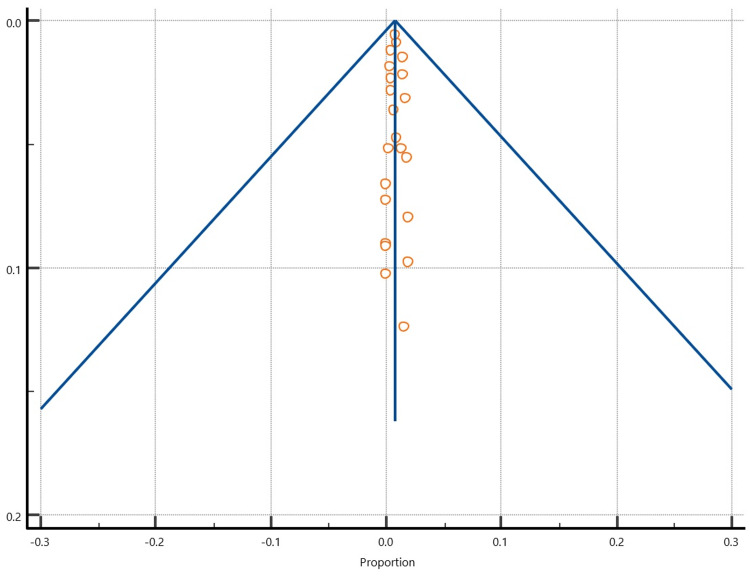
A funnel plot showing the symmetrical distribution of the plotted deep infection data

**Figure 7:**
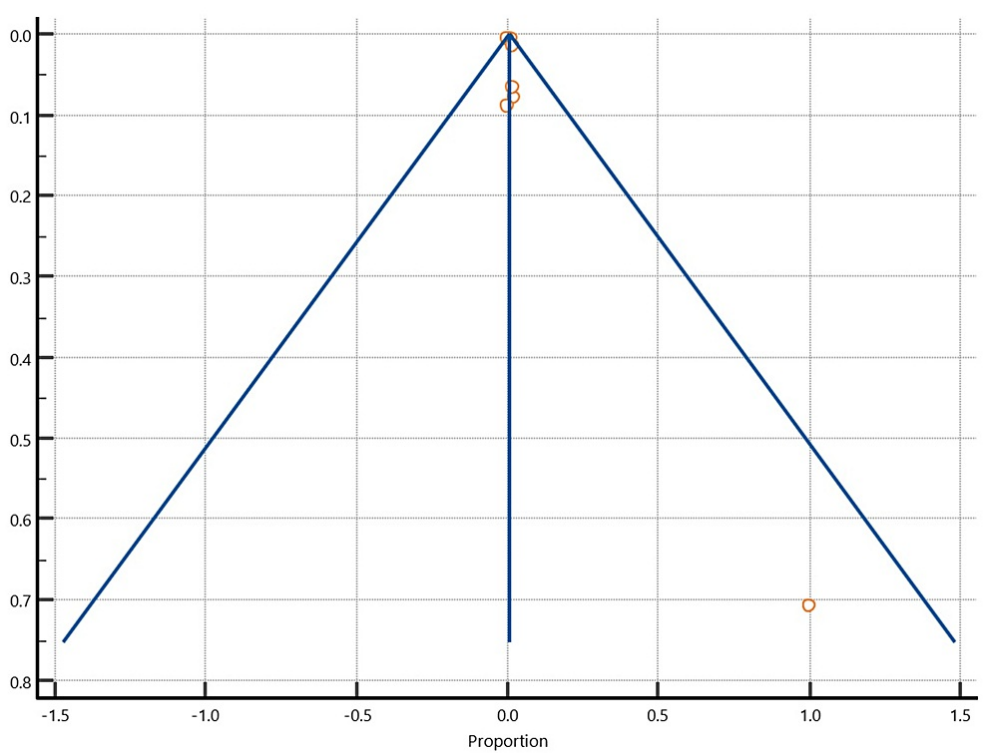
A funnel plot showing the symmetrical distribution of the plotted deep infection data

There was significant heterogeneity (I2>50%) in all analyses (Table 5). Visual inspection of funnel plots to detect publication bias (Figures 5-7) reveals a symmetrical distribution of the plotted data.

**Table 5:** Inter-study heterogeneity and publication bias

Parameter	Superficial infection	Deep infection	Unspecified
Q chi2	62.1893	82.3301	275.0070
DF {degrees of freedom}	13	21	7
Significance level	P < 0.0001	P < 0.0001	P < 0.0001
I^2^ (inconsistency)	79.10%	74.49%	97.45%
95% CI for I^2^	65.58 to 87.30	61.31 to 83.18	96.34 to 98.23
Egger's test			
Intercept	0.06168	0.1816	2.9529
95% CI	-1.8137 to 1.9371	-1.0893 to 1.4525	-4.0700 to 9.9759
Significance level	P = 0.9441	P = 0.7687	P = 0.3432
Begg's test			
Kendall's Tau	0.03297	0.09091	0.03704
Significance level	P = 0.8695	P = 0.5537	P = 0.8979

Discussion

The benefits of bilateral TKA performed under a single anesthetic regimen over bilateral arthroplasty performed in two stages include encouraging symmetrical rehabilitation of both knees, limiting invasive surgical procedures and anesthesia to a single event, possibly reducing the length of hospital stay, and, consequently, hospital costs related to TKA [4,5,17].

Our study aimed to estimate the prevalence of SSI following simBTKA by systematically reviewing current publications with relevant data. After a thorough screening, 30 studies were included in our meta-analysis, with a total population of 118,502 patients (237,004 knees). The pooled prevalence of superficial infection, deep infection, and unspecified SSI was estimated to be 0.86% (95%, CI: 0.62-1.13%), 0.84% (95%, CI: 0.64-1.05%), and 1.18% (95%, CI: 0.45-2.27%), respectively. There was significant heterogeneity (I2 > 50%) in all analyses. Furthermore, inspecting funnel plots revealed a symmetrical distribution of plotted data.

The safety of simBTKA has been thoroughly investigated by Malahias et al. [18] and a meta-analysis by Liu et al. [19]. According to Malahias et al., there is some evidence that simBTKA is just as safe as staged BTKA (staBTKA) in trials with patients with similar baseline demographics [18].

Upon comparing simBTKA with staBTKA, some studies have found fewer complications, such as infections, with simBTKA having lower rates of infection than staBTKA [8,20]. This finding is partially consistent with that of a recent meta-analysis that found that superficial infections occurred more frequently after staBTKA than after single anesthetic BTKA; however, periprosthetic infections occurred at similar rates [21]. The majority of single-anesthetic BTKA studies, however, do not differentiate between simBTKA and staBTKA; therefore, it is impossible to assume that the results and costs of both treatments will be similar [22]. As a result, few of these studies have provided sufficient data to compare the complication rates of simBTKA and staBTKA.

It is essential, however, to recognize that a staged operation postpones the full advantages of bilateral TKA until after the completion of both procedures. Additionally, when TKA is performed using a staged approach, a painful contralateral knee makes effective postoperative rehabilitation more challenging in patients with bilateral knee osteoarthritis and substantial flexion contractures. Increased stress in the contralateral knee after TKA is, therefore, believed to accelerate the development of osteoarthritis in the knee [23].

Recommendations

We recommend that further studies be conducted to provide higher-quality evidence to assess infection rates in simBTKA and its trends.

## Conclusions

From this meta-analysis, we reveal that the rates of infection following simBTKA are relatively low but heterogeneous, as the data shows marked variability. Superficial infections were more common than deep infections; however, there was a small difference in their prevalence. However, since the reliability of our findings was limited owing to the significant heterogeneity. 

## References

[1] Wales N (2017). National Joint Registry for England, Wales and Northern Ireland. 14th Annual Report. https://www.hqip.org.uk/resource/national-joint-registry-14th-annual-report-2017/.

[2] Klit J, Jacobsen S, Rosenlund S, Sonne-Holm S, Troelsen A (2014). Total knee arthroplasty in younger patients evaluated by alternative outcome measures. J Arthroplasty.

[3] Noble PC, Conditt MA, Cook KF, Mathis KB (2006). The John Insall Award: Patient expectations affect satisfaction with total knee arthroplasty. Clin Orthop Relat Res.

[4] Cohen RG, Forrest CJ, Benjamin JB (1997). Safety and efficacy of bilateral total knee arthroplasty. J Arthroplasty.

[5] Gradillas EL, Volz RG (1979). Bilateral total knee replacement under one anesthetic. Clin Orthop Relat Res.

[6] Hardaker WT Jr, Ogden WS, Musgrave RE, Goldner JL (1978). Simultaneous and staged bilateral total knee arthroplasty. J Bone Joint Surg Am.

[7] Jones CA, Beaupre LA, Johnston DW, Suarez-Almazor ME (2007). Total joint arthroplasties: current concepts of patient outcomes after surgery. Rheum Dis Clin North Am.

[8] Poultsides LA, Memtsoudis SG, Vasilakakos T (2013). Infection following simultaneous bilateral total knee arthroplasty. J Arthroplasty.

[9] Malinzak RA, Ritter MA, Berend ME, Meding JB, Olberding EM, Davis KE (2009). Morbidly obese, diabetic, younger, and unilateral joint arthroplasty patients have elevated total joint arthroplasty infection rates. J Arthroplasty.

[10] Meehan JP, Danielsen B, Tancredi DJ, Kim S, Jamali AA, White RH (2011). A population-based comparison of the incidence of adverse outcomes after simultaneous-bilateral and staged-bilateral total knee arthroplasty. J Bone Joint Surg Am.

[11] Pulido L, Ghanem E, Joshi A, Purtill JJ, Parvizi J (2008). Periprosthetic joint infection: the incidence, timing, and predisposing factors. Clin Orthop Relat Res.

[12] Page MJ, McKenzie JE, Bossuyt PM (2021). The PRISMA 2020 statement: an updated guideline for reporting systematic reviews. BMJ.

[13] Ma T, Tu YH, Xue HM, Wen T, Cai MW (2015). Clinical outcomes and risks of single-stage bilateral unicompartmental knee arthroplasty via Oxford phase III. Chin Med J (Engl).

[14] Tsay EL, Grace TR, Vail T, Ward D (2019). Bilateral simultaneous vs staged total knee 321 arthroplasty: minimal difference in perioperative risks. J Arthroplasty.

[15] Liu TK, Chen SH (1998). Simultaneous bilateral total knee arthroplasty in a single procedure. Int Orthop.

[16] Chan WC, Musonda P, Cooper AS, Glasgow MM, Donell ST, Walton NP (2009). One-stage versus two-stage bilateral unicompartmental knee replacement: a comparison of immediate post-operative complications. J Bone Joint Surg Br.

[17] Jankiewicz JJ, Sculco TP, Ranawat CS, Behr C, Tarrentino S (1994). One-stage versus 2-stage bilateral total knee arthroplasty. Clin Orthop Relat Res.

[18] Malahias MA, Gu A, Adriani M, Addona JL, Alexiades MM, Sculco PK (2019). Comparing the safety and outcome of simultaneous and staged bilateral total knee arthroplasty in contemporary practice: a systematic review of the literature. J Arthroplasty.

[19] Liu L, Liu H, Zhang H, Song J, Zhang L (2019). Bilateral total knee arthroplasty: Simultaneous or staged? A systematic review and meta-analysis. Medicine (Baltimore).

[20] Odum SM, Troyer JL, Kelly MP, Dedini RD, Bozic KJ (2013). A cost-utility analysis comparing the cost-effectiveness of simultaneous and staged bilateral total knee arthroplasty. J Bone Joint Surg Am.

[21] Xu C, Qu P, Deng T, Bell K, Chen J (2019). Does simultaneous bilateral total joint arthroplasty increase deep infection risk compared to staged surgeries? A meta-analysis. J Hosp Infect.

[22] Hu J, Guo DM, Lü Z, Liu J, Yu XL, Zhang ZN (2008). The clinical comparison of simultaneous bilateral total knee arthroplasty in treatment of osteoarthritis. J Naning Med Univ.

[23] Meehan JP, Monazzam S, Miles T, Danielsen B, White RH (2017). Postoperative stiffness requiring manipulation under anesthesia is significantly reduced after simultaneous versus staged bilateral total knee arthroplasty. J Bone Joint Surg Am.

[24] Bini SA, Khatod M, Inacio MC, Paxton EW (2014). Same-day versus staged bilateral total knee arthroplasty poses no increase in complications in 6672 primary procedures. J Arthroplasty.

[25] Bohm ER, Molodianovitsh K, Dragan A (2016). Outcomes of unilateral and bilateral total knee arthroplasty in 238,373 patients. Acta Orthop.

[26] Bolognesi MP, Watters TS, Attarian DE, Wellman SS, Setoguchi S (2013). Simultaneous vs staged bilateral total knee arthroplasty among Medicare beneficiaries, 2000-2009. J Arthroplasty.

[27] Chen JY, Lo NN, Jiang L (2013). Simultaneous versus staged bilateral unicompartmental knee replacement. Bone Joint J.

[28] Chua HS, Whitehouse SL, Lorimer M, De Steiger R, Guo L, Crawford RW (2018). Mortality and implant survival with simultaneous and staged bilateral total knee arthroplasty experience from the Australian Orthopaedic Association National Joint Replacement Registry. J Arthroplasty.

[29] Courtney PM, Melnic CM, Alosh H, Shah RP, Nelson CL, Israelite CL (2014). Is bilateral total knee arthroplasty staged at a one-week interval safe? A matched case control study. J Arthroplasty.

[30] Feng S, Yang Z, Sun JN (2019). Comparison of the therapeutic effect between the simultaneous and staged unicompartmental knee arthroplasty (UKA) for bilateral knee medial compartment arthritis. BMC Musculoskelet Disord.

[31] Gill SD, Hill-Buxton LM, Gwini SM (2020). Simultaneous (two-surgeon) versus staged bilateral knee arthroplasty: an observational study of intraoperative and post-operative outcomes. ANZ J Surg.

[32] Hadley S, Day M, Schwarzkopf R, Smith A, Slover J, Zuckerman J (2017). Is simultaneous bilateral total knee arthroplasty (BTKA) as safe as staged BTKA?. Am J Orthop (Belle Mead NJ.

[33] Hooper GJ, Hooper NM, Rothwell AG, Hobbs T (2009). Bilateral total joint arthroplasty: the early results from the New Zealand National Joint Registry. J Arthroplasty.

[34] Houdek MT, Wyles CC, Watts CD, Wagner ER, Sierra RJ, Trousdale RT, Taunton MJ (2017). Single-anesthetic versus staged bilateral total hip arthroplasty: a matched cohort study. J Bone Joint Surg Am.

[35] Hutchinson JR, Parish EN, Cross MJ (2006). A comparison of bilateral uncemented total knee arthroplasty: simultaneous or staged?. J Bone Joint Surg Br.

[36] Lindberg-Larsen M, Jørgensen CC, Husted H, Kehlet H (2015). Early morbidity after simultaneous and staged bilateral total knee arthroplasty. Knee Surg Sports Traumatol Arthrosc.

[37] Lindberg-Larsen M, Pitter FT, Husted H, Kehlet H, Jørgensen CC (2019). Simultaneous vs staged bilateral total knee arthroplasty: a propensity-matched case-control study from nine fast-track centres. Arch Orthop Trauma Surg.

[38] Memtsoudis SG, Ma Y, González Della Valle A, Mazumdar M, Gaber-Baylis LK, MacKenzie CR, Sculco TP (2009). Perioperative outcomes after unilateral and bilateral total knee arthroplasty. Anesthesiology.

[39] Namba RS, Inacio MC, Paxton EW (2012). Risk factors associated with surgical site infection in 30,491 primary total hip replacements. J Bone Joint Surg Br.

[40] Ritter MA, Harty LD, Davis KE, Meding JB, Berend M (2003). Simultaneous bilateral, staged bilateral, and unilateral total knee arthroplasty. A survival analysis. J Bone Joint Surg Am.

[41] Seo JG, Lee BH, Moon YW, Chang MJ, Park SH (2014). Disparate postoperative results in the first and second knees on simultaneous bilateral total knee arthroplasty. J Arthroplasty.

[42] Seol JH, Seon JK, Song EK (2016). Comparison of postoperative complications and clinical outcomes between simultaneous and staged bilateral total knee arthroplasty. J Orthop Sci.

[43] Sheth DS, Cafri G, Paxton EW, Namba RS (2016). Bilateral simultaneous vs staged total knee arthroplasty: a comparison of complications and mortality. J Arthroplasty.

[44] Sobh AH, Siljander MP, Mells AJ, Koueiter DM, Moore DD, Karadsheh MS (2018). Cost analysis, complications, and discharge disposition associated with simultaneous vs staged bilateral total knee arthroplasty. J Arthroplasty.

[45] Spicer E, Thomas GR, Rumble EJ (2013). Comparison of the major intraoperative and postoperative complications between unilateral and sequential bilateral total knee arthroplasty in a high-volume community hospital. Can J Surg.

[46] Triantafyllopoulos GK, Memtsoudis SG, Zhang W, Ma Y, Sculco TP, Poultsides LA (2016). Same-day surgery does not increase deep infection risk in bilateral total hip arthroplasty patients. J Arthroplasty.

[47] Wyles CC, Robinson WA, Maradit-Kremers H, Houdek MT, Trousdale RT, Mabry TM (2019). Cost and patient outcomes associated with bilateral total knee arthroplasty performed by 2-surgeon 324 teams vs a single surgeon. J Arthroplasty.

[48] Yoon HS, Han CD, Yang IH (2010). Comparison of simultaneous bilateral and staged bilateral total knee arthroplasty in terms of perioperative complications. J Arthroplasty.

